# Exploration of Noncoding Sequences in Metagenomes

**DOI:** 10.1371/journal.pone.0059488

**Published:** 2013-03-25

**Authors:** Fabián Tobar-Tosse, Adrián C. Rodríguez, Patricia E. Vélez, María M. Zambrano, Pedro A. Moreno

**Affiliations:** 1 Colombian Center for Genomics and Bioinformatics of Extreme Environments Gebix, Bogota, Colombia; 2 Department of Biology – FACNED-Universidad del Cauca, Popayán, Colombia; 3 Corporación CorpoGen, Bogotá, Colombia; 4 School of Computing and Systems Engineering - Universidad del Valle, Cali, Colombia; 5 PanAmerican Bioinformatics Institute, Santa Marta, Magdalena, Colombia; Albert Einstein College of Medicine, United States of America

## Abstract

Environment-dependent genomic features have been defined for different metagenomes, whose genes and their associated processes are related to specific environments. Identification of ORFs and their functional categories are the most common methods for association between functional and environmental features. However, this analysis based on finding ORFs misses noncoding sequences and, therefore, some metagenome regulatory or structural information could be discarded. In this work we analyzed 23 whole metagenomes, including coding and noncoding sequences using the following sequence patterns: (G+C) content, Codon Usage (Cd), Trinucleotide Usage (Tn), and functional assignments for ORF prediction. Herein, we present evidence of a high proportion of noncoding sequences discarded in common similarity-based methods in metagenomics, and the kind of relevant information present in those. We found a high density of trinucleotide repeat sequences (TRS) in noncoding sequences, with a regulatory and adaptive function for metagenome communities. We present associations between trinucleotide values and gene function, where metagenome clustering correlate with microorganism adaptations and kinds of metagenomes. We propose here that noncoding sequences have relevant information to describe metagenomes that could be considered in a whole metagenome analysis in order to improve their organization, classification protocols, and their relation with the environment.

## Introduction

Metagenomes represent a gold mine for biology, biomedicine, and biotechnology. Their studies have opened a window to find new products and environmental solutions, as well as to define relevant biological and ecological knowledge regarding the microorganisms. Most metagenomic data published has revealed new insights about the microbial world itself. Frequently, the study of metagenomes begins by decoding information in assembled or unassembled sequences, being the principal goal to analyze the genomic composition, functional dynamics, and biodiversity, which can be accomplished by different methods of prediction and comparison. Nowadays, metagenomic studies have revealed dependence among functional features, pathways, or biological processes, among metagenome niches [1]–[3]; for instance, some genes, metabolic pathways, and genomic features are associated to conditions of the environment studied [4]. These characteristics are the result of studying only the coding sequence depending on ORF predictions [5], leaving aside the noncoding sequences (NCS). Interestingly, the proportion of NCS in some metagenomes is up to ∼21% [6], which in big metagenomes could exclude many significant sequences.

The NCS in a metagenome could correspond to regulatory elements in prokaryotic or simple eukaryotic organisms [7]. However, there are other elements in NCS with structural or organizational genome function like repetitive DNA, that in some free-living bacteria are necessary for homologous DNA recombination and rearrangements [8]. Additionally, when a metagenome has a high amount of eukaryotic microorganisms, repetitive DNA is highly abundant and NCS increase due to their larger genomes and lower gene density [9]. Thus, different elements related to genome structure and regulation of metagenomes could be defined by exploring NCS.

Different methods have been used to search information in NCS in genomes and metagenomes, for example, identification of ribo-switches, noncoding RNA, or transcription factors in microbial genomes [10]–[12]. The most successful approaches to analyze these sequences are supported by sequence-based methods, not by sequence similarity-based methods like BLAST [13]. These sequence-based approaches analyze both coding and NCS from a different perspective, and not from comparisons [5]. In microbial genomics, sequence-based methods work by defining sequence patterns as (G+C) content, noncoding RNA, codon usage, di, tetra, or pentanuclotide frequencies [14]. These strategies can be used to identify regularities among microorganisms, for example, the existing relationship between trinucleotide frequencies and fingerprinting of geographic origins of *Mycobacterium tuberculosis* [15]. In contrast, the application of sequence-based methods in metagenomics has allowed comparison of organisms based on structural patterns, type of tetranucleotide frequencies [14], [16], and assignments of taxonomic groups in metagenome samples based on noncoding elements [17]. They have also been used to define new features in coding and NCS such as structural RNA organization in archaea [18], or for metagenome binning based on *l*-mer composition [19].

The (G+C) content, codon usage, and tetranucleotide frequencies have been the most successful and most studied sequence patterns in metagenomics [14], [16], [18]; however, codon and tetranucleotides are directly associated with coding sequences [20], they are not useful for analysis of NCS or whole metagenome studies. In this work, we evaluated trinucleotide usage pattern in conjunction with the whole metagenome composition and their biological significance. We analyzed the coding and NCS from several metagenomes deposited at the *DOE Join Genome Institute JGI* (http://www.jgi.doe.gov/), by making comparisons of structural and functional profiles defined by sequence and similarity-based methods.

## Results

In this work we examine four main approaches to study the noncoding sequences in twenty three metagenomes with different environmental conditions.

### Metagenome Dataset and Noncoding Sequences

Table 1 shows the metagenomes and their sizes. DOE JGI classifies these metagenomes as *environmental (Env)*, *host-associated (HAs)*, and *engineered (Eng)* based on the type of ecosystem, host phylogeny, and function [21]. One important feature related to this classification is the size of the metagenomes, where those with more than 17 Mbp were defined as dense, and those with less than 9.4 Mbp were defined as non-dense. For example *soil microbial communities from a Minnesota Farm* (SMF) represents a dense metagenome, and *Olavius algarvensis endosymbiont* (OAEM) represents a non-dense metagenome. It is important to consider that in non-dense metagenomes it is common to find large DNA sequences (more than 1 Kb) that compensate for the few sequences and allows application of the sequence-based approaches.

**Table 1:** DOE JGI metagenomes classification and number of sequences analyzed.

DOE JGI Metagenome Classification					Number ofSequences	Size Mpb	Category based on size
Environmental	Terrrestrial	soil	**SMF**	Soil microbial communities from Minnesota farm	**126821**	144.6	Dense
	Aquatic	Thermal Springs	**OHSY**	Obsidian hot spring Yellowstone	**3442**	4.3	Non-Dense
		Marine	**AOM**	Anaerobic methane oxidation (AOM) community from Eel River Basin sediment, California	**60**	2.1	Non-Dense
		Freshwater	**AMD**	Acid Mine Drainage (Iron Mountain)	**1183**	10.8	Non-Dense
			**MLWSFD**	Methylotrophic community from Lake Washington sediment	**71686**	57.6	Dense
			**MLWSF**	Methylotrophic community from Lake Washington sediment F	**22475**	17.6	Non-Dense
			**MLWSME**	Methylotrophic community from Lake Washington sediment ME	**62214**	52.2	Dense
			**MLWSMO**	Methylotrophic community from Lake Washington sediment MO	**59278**	50.2	Dense
			**MLWSML**	Methylotrophic community from Lake Washington sediment ML	**36774**	37.2	Dense
			**UCG**	Uranium Contained Groundwater FW106	**5914**	9.4	Non-Dense
Host-Associated	Host-Associated	Host-Associated	**AVCYNL**	Archaeal virus community from Yellowstone HotSprings (Nymph Lake)	**953**	0.9	Non-Dense
			**AVCYCH**	Archaeal virus community from Yellowstone HotSprings (Crater Hills)	**1540**	1.7	Non-Dense
			**EMR**	Endophytic microbiome from rice	**57219**	46.7	Dense
			**MEGM**	Macropus eugenii gut microbiome	**53388**	53.9	Dense
			**OAEMD1**	Olavius algarvensis endosymbiont metagenome D1	**226**	13.5	Non-Dense
			**OAEMD4**	Olavius algarvensis endosymbiont metagenome D4	**172**	6.4	Non-Dense
			**OAEMG1**	Olavius algarvensis endosymbiont metagenome G1	**10**	0.1	Non-Dense
			**OAEMG3**	Olavius algarvensis endosymbiont metagenome G3	**22**	4.6	Non-Dense
Engineered	Engineered	Engineered	**ANASDB**	ANAS dechlorinating bioreactor (Sample 196)	**26293**	41.1	Dense
			**ADC**	Aquatic dechlorinating community (KB-1) (Sample 10166)	**24990**	29.9	Dense
			**SAU**	Sludge Australian	**22363**	52.9	Dense
			**SUS**	Sludge US	**31606**	56.4	Dense
			**WTDBR**	Wastewater Terephthalate-degrading communitiesfrom Bioreactor	**52342**	59.6	Dense

**Figure 1:**
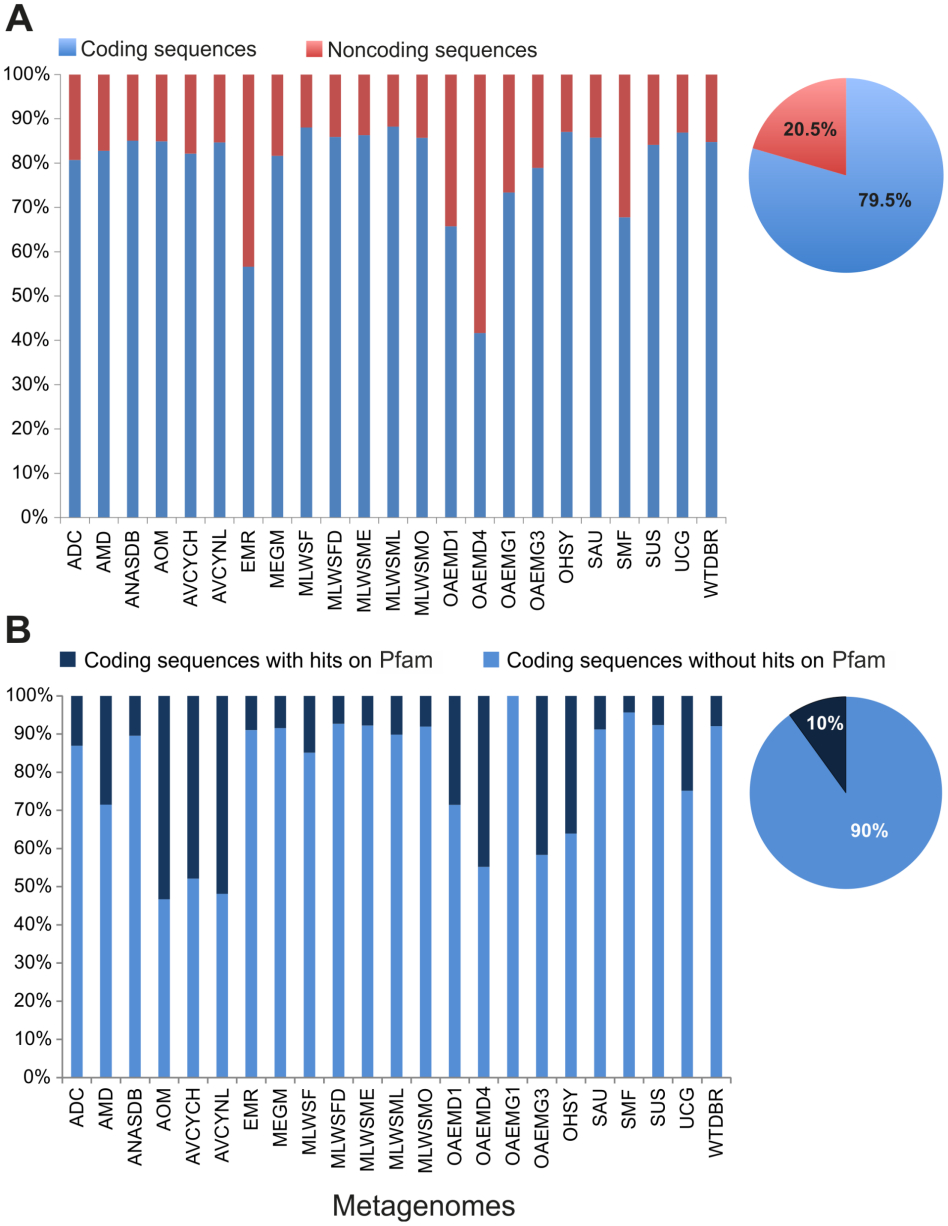
Use of coding and noncoding sequences. **A.** Proportion of coding and noncoding metagenome sequences based on ORF prediction. **B.** Proportion of coding sequence with hits against Pfam database.

We identified the proportion of coding and NCS for each metagenome (Figure 1A), finding a smaller proportion of NCS (∼20.5%) that contrasts with a significant amount of coding sequences (∼79.5%) to be analyzed. Six metagenomes had more than 20.5% of NCS (EMR, OAMD1, OAMD4, OAMDG1, OAMED3 and SMF). From this global landscape, the association between NCS and environmental conditions for some metagenomes, like *Endophytic microbiome from rice* (EMR) and *Olavius algarvensis endosymbiont metagenomes* (OAEM), is exposed, showing a relation between a high proportion of NCS and the *HAs* metagenomes. However, expected associations like dense metagenomes with a high proportion of NCS were discarded because dense metagenomes like SMF or *Methylotrophic community from Lake Washington sediment* (MLWSF) have less NCS than others.

The association of functions to predicted ORFs or coding sequences via BLAST programs is a similarity-based method common in metagenomics that allows understanding the functional complexity of the metagenomes. Upon identifying which of the predicted coding sequences have associations with functional information (Pfam categories) [22], we found that not all coding sequences had functional assignments and, therefore, could not be used for metagenome functional description. The proportion of predicted ORFs associated to Pfam models was very low ∼10% (Figure 1B), which in the context of all metagenomes can be represented as ∼13% of coding sequences *with functional assignments*, and ∼66.5% of coding sequences *without functional assignments*. Interestingly, there were non-dense metagenomes with more functional associations than dense metagenomes, as was the case for *Anaerobic methane oxidation* (AOM) and *Archaeal virus community from Yellowstone* (AVCY) metagenomes that had more than 40% of coding sequences with functional associations. In contrast, SMF or MLWS (dense metagenomes) had less than 10% of the coding sequences with functional associations. Finally, there were no associations between dense and non-dense metagenomes and coding sequences because the proportions of coding sequences with functional associations varied among all metagenomes.

### Metagenome Description by Sequence-based Methods

**Figure 2:**
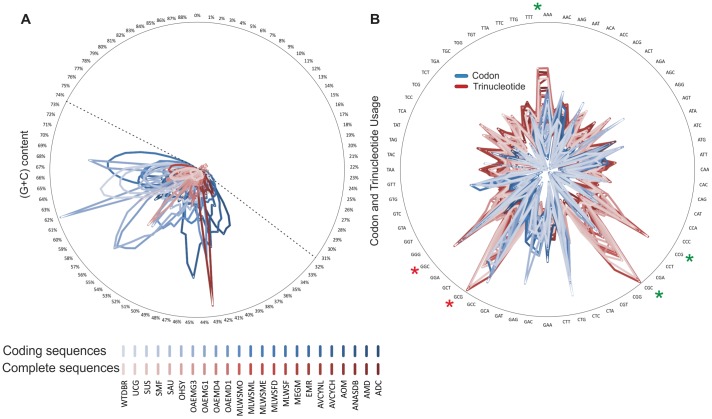
Sequence patterns defined. **A.** (G+C) content distribution in metagenomes. **B.** Codon and trinucleotide usage. (Blue: Coding sequences; Red: Entire sequences).

The sequence patterns used in this sequence-based approach exposed features associated with composition and organization of DNA sequences. For composition, (G+C) content was the first measure used to characterize coding and complete (coding and noncoding) metagenome sequences (Table S2), radially plotted in Figure 2A. This pattern showed different ranges of distribution for coding and complete sequences, in which small peaks in the radial distribution represent non-specific (G+C) content and large peaks indicate a tendency to high (G+C) content. This analysis revealed that coding sequences (blue peaks) had some specific (G+C) content peaks, for example, around 68, 62, 56, and 44.5%, while the complete sequences (red peaks) only had one (G+C) content peak around 43% given by AOM metagenome, which corresponds to a high proportion of (G+C) content for noncoding elements.

A second measure to characterize NCS in the metagenomes was implemented using the codon (for coding sequences) and trinucleotide (for complete sequences) contents (Figure 2B, Table S3). The radial distribution of these patterns clearly showed similarities and differences between coding and complete sequences. According to this, there are similar codon and trinucleotide compositions with similar usage tendency like GGC or GCG (red asterisk), which shows a relationship between coding and NCS. That means that the codons and triplets might be used simultaneously for protein synthesis and likely for promoter regions. On the other hand, the high uses of trinucleotide compositions different from codons in complete sequences are the most relevant feature in this work. This is because the trinucleotides CGC, CCG, TTT, and AAA are highly used in NCS (green asterisks), which may be a relevant structural feature of metagenomes, like that observed for TRS. Interestingly, these tendencies or high use of trinucleotides were observed for aquatic metagenomes (UCG, MLWSF) and might be associated with a new environmental-dependent feature for those metagenomes.

### Metagenome Description by Similarity-based Methods

**Figure 3:**
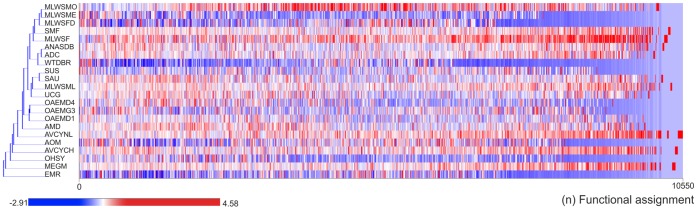
Functional analysis of coding sequences from metagenomes. Identification of metagenome clustering according to functional assignments based on Pfam models. The color bar indicates frequency of functional category from low (blue) to high (red).

Similarity-based methods were applied to compare functional and structural features. The coding sequences with Pfam [22] associations were studied to identify relevant functions in metagenomes, but are not described further because functional environment-dependent features have already been described extensively [1]–[3]. A comparative file called “functional profile” was generated for all metagenomes, which has all the functional assignments and their frequency of use in each metagenome. This profile was analyzed by hierarchical clustering, as shown in Figure 3 (Table S4). This approach allowed us to define clustering of metagenomes according to functional assignments. Herein, we identified regularities among the kinds of metagenomes and their sets of functions. For example, clusters were formed with the metagenomes from the *Methylotrophic community from Lake Washington sediment* (MLWSMO, MLWSME, MLWSFD, MLWSF) or from *Olavius algarvensis endosymbiont* (OAEMD4, OAEMG3, OAEMD1), which are examples of specific niches with common sets of functions, whose microbial communities maintain similar sets of proteins related to the environment requirements or cell necessities. Interestingly, the metagenome SMF showed several common functions with the MLWS cluster, suggesting possible similarity in the microbial community and functional requirements in these soil and sediment ecosystems.

**Figure 4:**
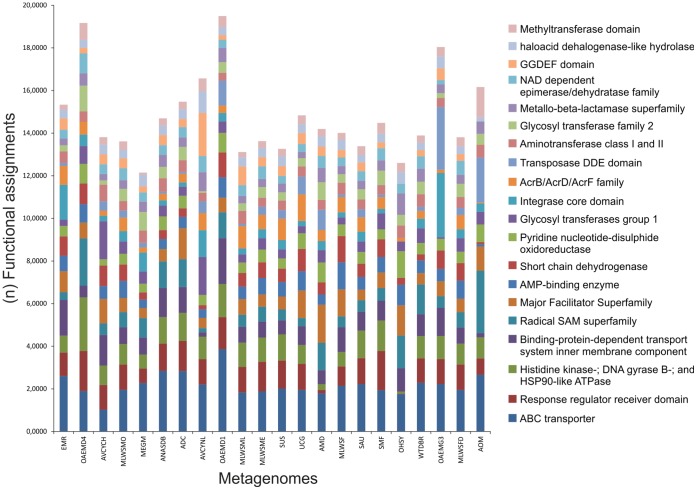
Common functional assignments among metagenomes. The size of the bars indicates the number per functional category, and the colors indicate the type of category.

The other metagenomes were arrayed in diverse clusters and involved a combination of *Env*, *HAs*, and *Eng* metagenomes, indicating that there are several common functions among these metagenomes. These common functions were selected and the most conserved functions were identified (Figure 4). As expected, these functional associations are related to cell viability as (catalytic and anabolic) enzymes, mobile element mechanisms, translocation of various substrates across membranes by ABC transporters, and phosphorylation-mediated switches by response regulator receiver domains (Table S5). These common functions show common dynamics among microorganisms from different environments, but not specific functions for each metagenome.

**Figure 5:**
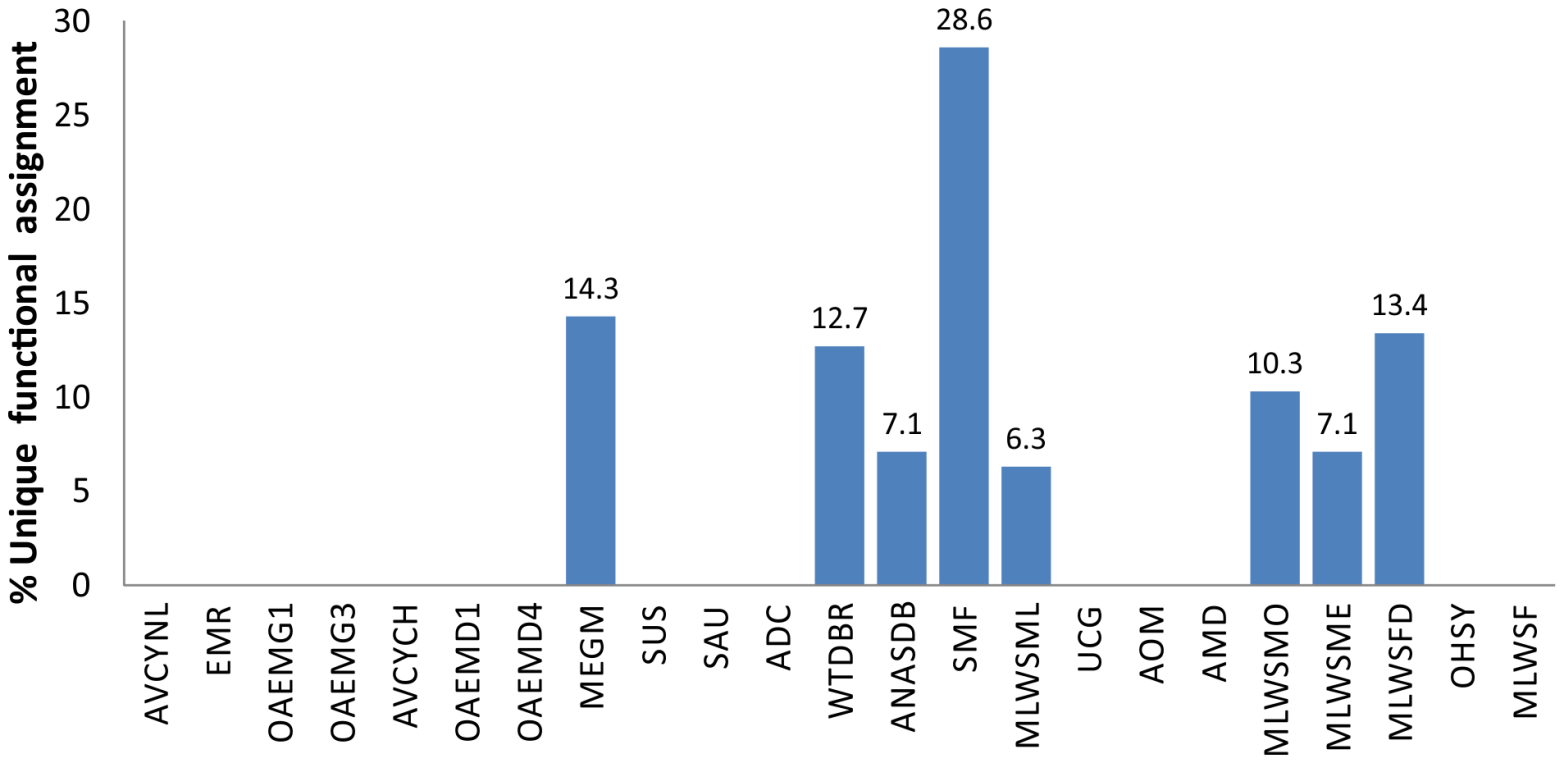
Proportion of functional assignments unique for each metagenome.

In order to identify the proportion of unique functional assignments for each metagenome, we used the functional profile to extract the number of unique assignments for each metagenome (Figure 5, Table S6). The result of this approach showed only 8 metagenomes with a unique set of functions. This feature was associated to specific adaptations in accordance with different niches or environmental conditions because these metagenomes are distributed in the three studied categories. A particular feature in the metagenomes from MLWS (Methylotrophic community from Lake Washington sediment) is revealed by the fact that four of the five metagenomes had unique sets of functions, not common to all, that could reflect metabolic adaptations for particular substrates in the same community, as has been proposed [23].

**Figure 6:**
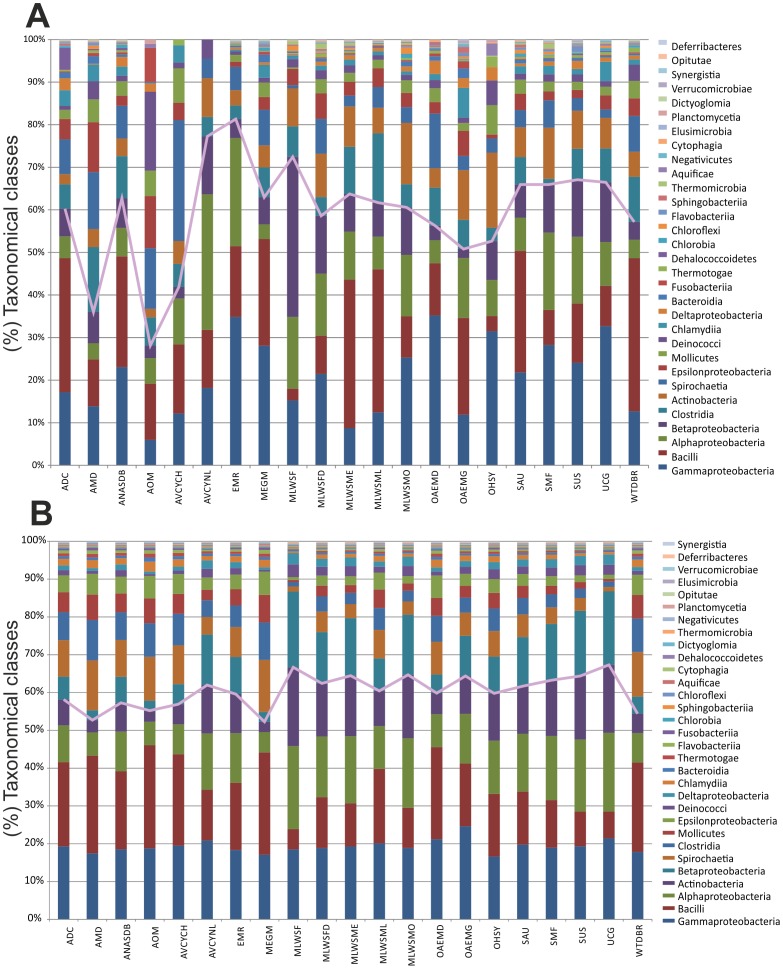
Proportion of NCS mapped to complete bacterial genomes. **A.** Distribution of taxonomical classes mapped in complete genomes with NCS. **B.** Distribution of taxonomical classes mapped in coding sequences with NCS.

Subsequently, we investigated whether metagenomic NCS were present in complete annotated genomes by examining the proportion of NCS mapped in available sequenced genomes. 4189 genomes and sets of coding sequences were challenged against the entire set of metagenomic NCS. Figure 6A represents the percentage of BLAST hits associated to 31 taxonomic classes. For most classes, a 60% were found in the first 4 taxonomic classes (Gammaproteobacteria, Bacilli, Alphaproteobacteria, and Betaproteobacteria). The remaining 40% involved the other taxonomic classes. Figure 6B, shows a similar behavior for the hits using coding sequences (Table S7).

### Functional and Structural Profiles

**Figure 7:**
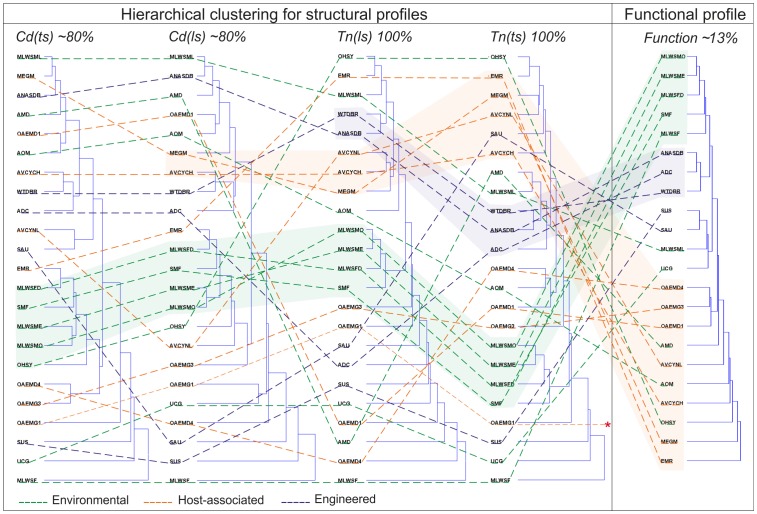
Hierarchical clustering trees. Representation of structural and functional profiles for *Env*, *HAs* and *Eng* metagenomes. The lines correspond to the metagenome category and the shaded sections correspond to conserved clustering organization of metagenomes among the trees. Asterisk indicates not functional associations for the OAEMG1 metagenome.

Finally, the trinucleotide and codon usages profiles *Tn(ls)*, *Tn(ts)*, *Cd(ls)*, and *Cd(ts)*, were calculated. These correspond to normalized values used to compare metagenomes, based on the length *(ls)* and number of triplets *(ts)* by sequence. These values defined four structural profiles and the Pfam assignments defined one functional profile (see Methods). The comparison of the functional and structural profiles was obtained by mean construction of hierarchical clustering trees (Figure 7). It is important to note, structural and functional profiles were based on different percentages of analyzed sequences since this depends on the method used. Sequence-based approaches defined (*Tn(ls)* and *Tn(ts)*) with 100%, and (*Cd(ls)* and *Cd(ts)*) with ∼80%; and the similarity-based approach used ∼13% of the sequences. In order to analyze the relevance of the structural patterns in terms of classifying the metagenomes, several comparisons were made between structural profiles trees and the functional profile tree. The *Env* and *HAs* metagenomes were organized in two clear clusters, showing patterns of organization that have been described by other authors [1]–[3]. These clusters were then used to compare them with the structural profiles trees (lines in Figure 7). Although the structural hierarchical trees differed in cluster distribution, some regularities were observed (fringe shaded), such as a clustering conservation in the categories *Env*, *HAs*, or *Eng* between the functional and *Tn(ts)* profiles for some metagenomes.

## Discussion

Here, we studied particular metagenomic features based on whole sequence analysis that includes noncoding elements, usually left out in standard methods in metagenomics. This means that only a subset of sequences is analyzed in metagenomes using the common method of ORF prediction where NCS are discarded or used only to improve methods in gene finding [24], [25]. In this work, seven relevant aspects will be discussed.

### The NCS from Several Metagenomes were Studied

The NCS are not well studied and are not used to identify functional or environment features in metagenomic analysis. However, the proportion of NCS is higher (∼20.5%) than that of coding sequences with Pfam assignments (∼10%) that are used commonly in metagenome functional analysis (Figure 1). Although these proportions can depend on the prediction methods, a similar proportion of NCS was defined previously for other metagenomes and by different programs [5]. Thus it is plausible to define these proportions of coding and NCS as particular feature of the metagenome composition. In addition, considering the proportion of NCS in prokaryotes ∼18% [26] and unicellular of simple eukaryotes ∼30% [27], these metagenomic NCS could harbor relevant information regarding the different microbial populations.

### A Wide Range of (G+C) Contents in Metagenomic NCS was Revealed by Sequence-base Methods

In microbial genomes NCS are involved in regulation and rearrangements of the genomic content, both of which are important for adaptation to changing environments [9]–[11]. These features can be related with sequence patterns in NCS that differ from those in coding sequences, and these are discriminatory elements for gene prediction [25]. This idea agrees with the sequence patterns presented in Figure 2A where there are evident differences in the range of distribution of the (G+C) content between coding and complete sequences, which could reflect abundant elements in NCS with a large range of compositions. Additionally, Figure 2A shows that all coding sequences are mainly distributed in a (G+C) range between 32 and 73% (dashed lines), where all metagenomes are located. This “range of life” seems to be flanked by sequences, rich in repetitions perhaps subjected to different processes of selection, adaptation, or environmental stress. In contrast, below 32% and above 73% there seems to be no complete metagenome. Analysis of all complete bacterial genomes deposited at the NCBI shows that below 13.5% and above 75% it is hardly possible to find any living organism (Table S8). (G+C) percentages <32% and >73% seem to be primarily occupied by organisms involved in symbiotic associations and intracellular life styles or by aerobic organisms, where (G+C) values are higher [28].

### An Abundant Number of TRS Elements were Found in NCS

The results obtained with the codon and trinucleotide usage (Figure 2B), indicate that the abundant elements in NCS are TRS (TTT, AAA, CGC, CGG, and CCG). The definition of TRS in metagenomes depends on the density and comparison with codons, because similar triplet density both in coding and NCS involves the same element; and the differences confer specific triplets to coding (as codon) or NCS as TRS. Accordingly, we have identified three relevant TRS (CGG, CGC, and CCG) by the high density and distribution across several metagenomes, mainly in UCG and MLWSF metagenomes. These TRS could be involved in adaptations and genetic susceptibility to variations [15], or they could be associated to noncoding RNA with a regulatory function in transcriptional processes [11]. Thus, the TRS represent simple sequence repeats, abundant in metagenomes and possibly involved in adaptation to different environmental conditions, as has been defined in prokaryotic genomes [29]. This idea still has to be explored more deeply.

### A Large Proportion of NCS is Present in Complete Genomes (Figure 6)

This can be discussed in two ways. One explanation might be that many sequenced bacterial organisms might be part of the microbiota of these metagenomes or related with, in the worse cases, pollution phenomenon. A further analysis with 16S rRNA might verify the presence of theses genomic classes identified by us. Another explanation might be related to lateral gene transfer.

### Functional Assignments are Related to Metagenome Sizes

Figures 3, 4, and 5 showed several typical behaviors of functional assignments per metagenome. This complex distribution (in Figure 4) seems to be related to the metagenome size. That is, there exists a strong relationship between the numbers of functional assignments and the metagenome size (R^2^ ∼0.91) (Table S1). For example, the SMF metagenome has the highest value, whereas the AVCYNL metagenome has the lowest one. This is because the SMF metagenome is environmental (soil), while the AVCYNL is host-associated, which might be expected, since this trend is observed for other related metagenomes. On the other hand, no evidence of functional pattern can be studied in the NCS by functional profiles, due to the fact there is not annotation for these sequences in the bacterial database. However, a diversity of functional elements, type of noncoding RNA (ncRNA), among others, has been identified in NCS as key players in gene regulation [30].

### Trinucleotide Patterns and Structural Profiles Help to Identify Features among Metagenomes and the Environment

In this work we carried out a whole metagenome analysis using coding and NCS and showed that NCS are significant and contain relevant information, such as the trinucleotide organization that in some of cases is common for several metagenomes. With the aim of comparing the metagenomes based on associated trinucleotides values, we propose *Tn(ls)*, *Tn(ts)*, *Cd(ls)*, and *Cd(ts)* as the structural profiles with the capacity to embrace all the trinucleotides *(Tn)* or codons *(Cd)*, and define a comparable value to each metagenome. An increase in any of these values means that specific trinucleotides are being used with high frequency in the metagenome; in contrast, low values indicate a non-conserved use of trinucleotides or codons. These patterns, which could help to identify features among metagenomes and correlations with environments, were used to make a classification of the metagenomes in a hierarchical tree (Figure 7). The relevance of this clustering organization lies in the proportion of metagenome sequence used for each profile, for example the *Tn(ts)* use 100% of the metagenome sequences, whereas the functional profile uses only ∼13% of them. As result, the use of *Tn(ts)* could capture regularities in the NCS. Here, we propose that the clustering similarities and differences of metagenomes based on *Tn(ts)* and functional profiles have biological meanings. The similarities these are related to conserved cellular mechanisms in coding sequences and NCS, like specific mechanisms of regulation for specific genes. In contrast, differences are related to conserved elements not present in functional profiles, but present in NCS, like TRS or ncRNAs [11]. These describe possible connections among microorganisms based on complex mechanism of regulation. The differences of clustering in structural profiles are directly related with the constants of normalization i) length (*ls*), and ii) number of trinucleotides or codons by sequence, *Tn(ts)* or *Cd(ts)*), where *Tn(ts)* is more precise to compare metagenomes according to the comparison with functional profile tree. This could be due to more changes in the NCS more than in coding sequences that conserve basic functions but also allow for a more dynamic genome. The clustering of the MLWSMO, MLWSME, MLWSFD, and SMF metagenomes across all the trees would indicate that the possible organization or patterns in the NCS could be connected to those protein motifs present in the coding sequences. This regularity is only revealed for *Env* metagenomes, which are not affected by drastic environmental fluctuations and allow a controlled organization, as a model for genetic exchange and adaptation [31], for example the temperature in archaeal organisms and the GC variations [32]. Thus, possible reorganization of genome elements in the NCS occurs less frequently in *Env* than in *HAs* where microorganisms need to adapt to the imposed and varying host cell conditions [27]. Finally, *Eng* metagenomes have no specific distribution or clustering of metagenomes, possibly because these communities are subjected to strong and different environmental pressures to carry out a great variety of functions required for specific adaptations and genomic rearrangements in each environment [33]. However, it would be important to identify elements that could lead to a possible connection, and be used in biotechnology.

### A Framework for Studying the Environmental Metagenomes is Proposed

**Figure 8:**
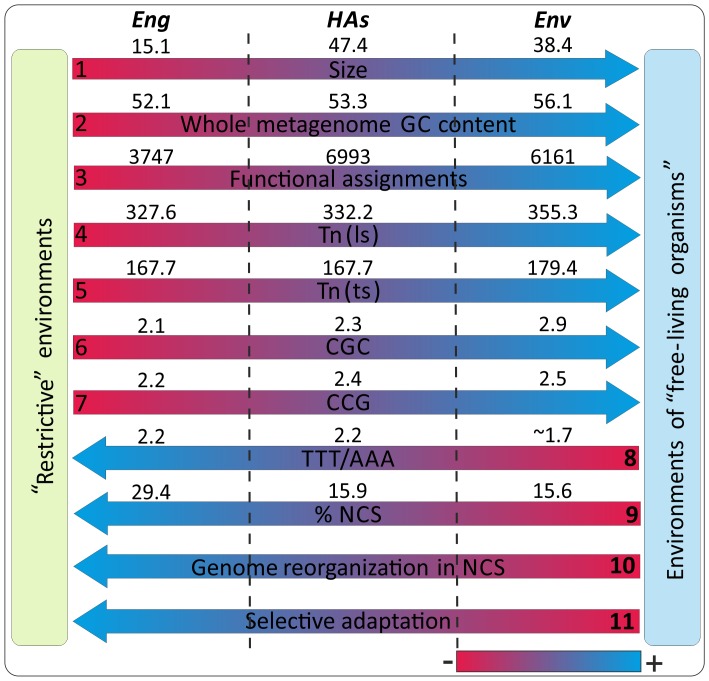
A metagenomic framework. At the top are shown, the three metagenomic categories. Averaged values per category for each parameter are shown above the arrows. The parameters (1–8) were calculated from complete metagenomes, parameter 9 was calculated from NCS (Table S1) and parameters 10 and 11 are behaviors inferred from literature [9]–[11].

All these results suggest a related metagenomic framework. Despite analyzing a small number of metagenomes, this sample allows us to identify some significant correlations and trends in the direction: *Eng* -> *HAs* -> *Env*. For that, some relevant features were examined and discussed (Figure 8, Table S1). Initially, the average (G+C) content per metagenomes category increases very little (from 52.5 to 56%), but this trend could only be relevant for aerobic organisms [27]. Nonetheless, the *Tn(ts)* and *Tn(ls)* usages are moderately correlated with the (G+C) contents (R^2^ ∼0.63). In terms of some specific triplets (CGC, CCG, TTT, and AAA) these relationships are considerably high (R^2^ ∼0.9, ∼0.95, ∼0.85, and ∼0.86, respectively). The number of functional assignments increases greatly and this is inversely related to the percentage of NCS, the abundance of TRS (especially for TTT and AAA), the reorganization of the genome NCS, and adaptation to the environment. These features by metagenomic category would be connected, thereby, to a larger number of NCS (rich in regulatory sequences and TRS) that might contribute to increase the number of genomic rearrangements and establish selective adaptation processes through the use of a smaller number of functional assignments. All these trends and directions seem to suggest a related framework of metagenomic parameters (or features) moving from “restrictive” environments to environments of “free-living organisms”.

In conclusion, the sequence-based methods, specifically *Tn(ts)*, effectively help to define regularities in the organization of the metagenomes and, second, the NCS can contain relevant information for metagenome classification and microorganism functional description that needs to be studied more deeply. Undoubtedly, the common functional environment-dependent features proposed by other authors could be associated to structural environment-dependent features. Consequently, environment-dependent features could be defined by the study of the whole metagenome. Thus, the proposed metagenomic framework only is possible taking into account all the information coded by complete metagenomes.

## Materials and Methods

Five methodological steps were followed in this study (Figure S1).

### 1. Metagenome Data Sets

A total of 23 metagenomes were downloaded from the metagenomics program at the DOE Joint Genome Institute JGI (http://www.jgi.doe.gov/) (Febrary 2010). Based on type of ecosystem, host phylogeny, and function, these metagenomes are classified as environmental (*Env*), host-associated (*HAs*), and engineered (*Eng*) [21]. The sequences downloaded correspond to DNA scaffolds as DOE-JGI presents the data, pre-cleaned. An additional cleaning was made by a python scripts to avoid sequences with ≤20 bp, and X (unknown) and N (unspecified) contents >25%.

### 2. Noncoding Sequence Identification and Sequence-based Methods

Coding and noncoding sequences were determined through ORF prediction with MetaGeneMark algorithm [24]. Three sets of data were defined to each metagenome: Coding sequences (ORF predictions), Complete sequences (Coding and Noncoding sequences) and Noncoding sequence (region of the sequences without ORF predictions). The sequence-based methods applied in this work involved the definition and analysis of three sequence patterns: (G+C) content, Codon Usage (Cd), and Trinucleotide usage bias (*Tn*), *Tn* according with [34]. These patterns were applied on coding and complete sequences conferring structural pattern values, defined by two assessments: i) Trinucleotide (Complete sequences) or codon (Coding sequence) values based on length *Tn(ls)* or *Cd(ls)* respectively, these were defined as the sum of trinucleotide usage frequencies (*Tn*) or codon usage frequencies (*Cd*), over the length of sequence (*l*)*:* “*Tn(ls) = Σ(Tn)/l*” or “*Cd(ls) = Σ(Cd)/l*”. And ii) Trinucleotide or Codon values based on the number of trinucleotides or codons by sequence, *Tn(ts)* or *Cd(ts)*, respectively. These were defined as the sum of trinucleotide usage frequencies (*Tn*) or codon usage frequencies (*Cd*), over the number of trinucleotides *n(Tn)* or codons *n(Cd):* “*Tn(ts) = Σ(Tn)/n(Tn)*” or “*Cd(ts) = Σ(Cd)/n(Cd)*”. These values above were organized in a comparative table named as ¨structural profiles (Table S3).

### 3. Functional Assignments and Similarity-based Methods

The peptides from predicted ORFs were assigned to a functional feature using BLASTP [13] (BLAST 2.2.25 release) methods as propose [35]. Pfam-A was used as local database (*February 2010 release, 11912 models in total available at* www.sanger.pfam.com) [22], and a cutoff: e-value < = 1e^−30^, identity > = 95%. The resulting Pfam assignments were integrated into a unique file named ¨functional profilë table (Table S4), which lists the Pfam models with a value for each model defined as the frequency of assigned sequences for each model by metagenome: “*ƒ(Pfam) = (Pfam_nq_)/N(Pfam)”*. Where *f(Pfam)* is the frequency of the Pfam model in the metagenome; *(Pfam_nq_)* are the number of BLAST queries assignments for the model, and *N(Pfam)* is the total number of Pfam models with associations in the metagenome. An additional approach was applied, related to blast searches of NCS in complete bacterial genomes to the association of any annotated function or taxonomy (BLASTn, e-value < = 1e^−10^).

### 4. Functional and Structural Profiles

Four structural profiles were made, two based on coding sequences (*Cd(ls)*, *Cd(ts)*), two based on complete sequences (*Tn(ls)*, *Tn(ts)*), and one functional profile based on functional associations. Those profiles are comparatives tables, which compares the 23 metagenomes. The functional and structural profiles were analyzed by hierarchical trees using the Hierarchical Cluster Explore tool (HCE) [36].

### 5. Metagenomic Framework

For each metagenome category (*Env*, *HAs*, and *Eng*), ten parameters (size, whole metagenome (G+C) content, functional assignments, *Tn(ls)*, *Tn(ts)*, CGC, CCG, TTT, AAA, and percentage of NCS) were averaged and calculated per metagenome and per metagenome category (Table S1). Coefficients of correlation were calculated by simple linear regression for some of those parameters.

## Supporting Information

Figure S1 **Flowchart of methodological steps.**
(TIF)

Table S1 This file contains several counts related with the sequences for each metagenome and the metagenome categories *Eng*, *HAs*, *Env*.
(XLS)

Table S2 This file contains the frequency of (G+C) contents for coding and complete sequences in 23 metagenomes.
(XLS)

Table S3 This file contains the structural profile for 64 triplets in 23 metagenomes.
(XLS)

Table S4 This file contains the functional profile for 23 metagenomes.
(XLS)

Table S5 This file contains the most representative functional assignments.
(XLS)

Table S6 This file contains the unique functional assignments for 23 metagenomes.
(XLS)

Table S7 This file contains the taxonomic classes from complete bacterial genomes associated to NCS, based on BLAST hits.
(XLS)

Table S8 This file contains the (G+C) contents measured for complete bacterial genomes from the NCBI.
(XLS)
