# Supplementary figures and images for: Exploration of Noncoding Sequences in Metagenomes
Source: PLoS One. 2013 Mar 25;8(3):e59488. doi: 10.1371/journal.pone.0059488 (PMC3607601; doi:10.1371/journal.pone.0059488)

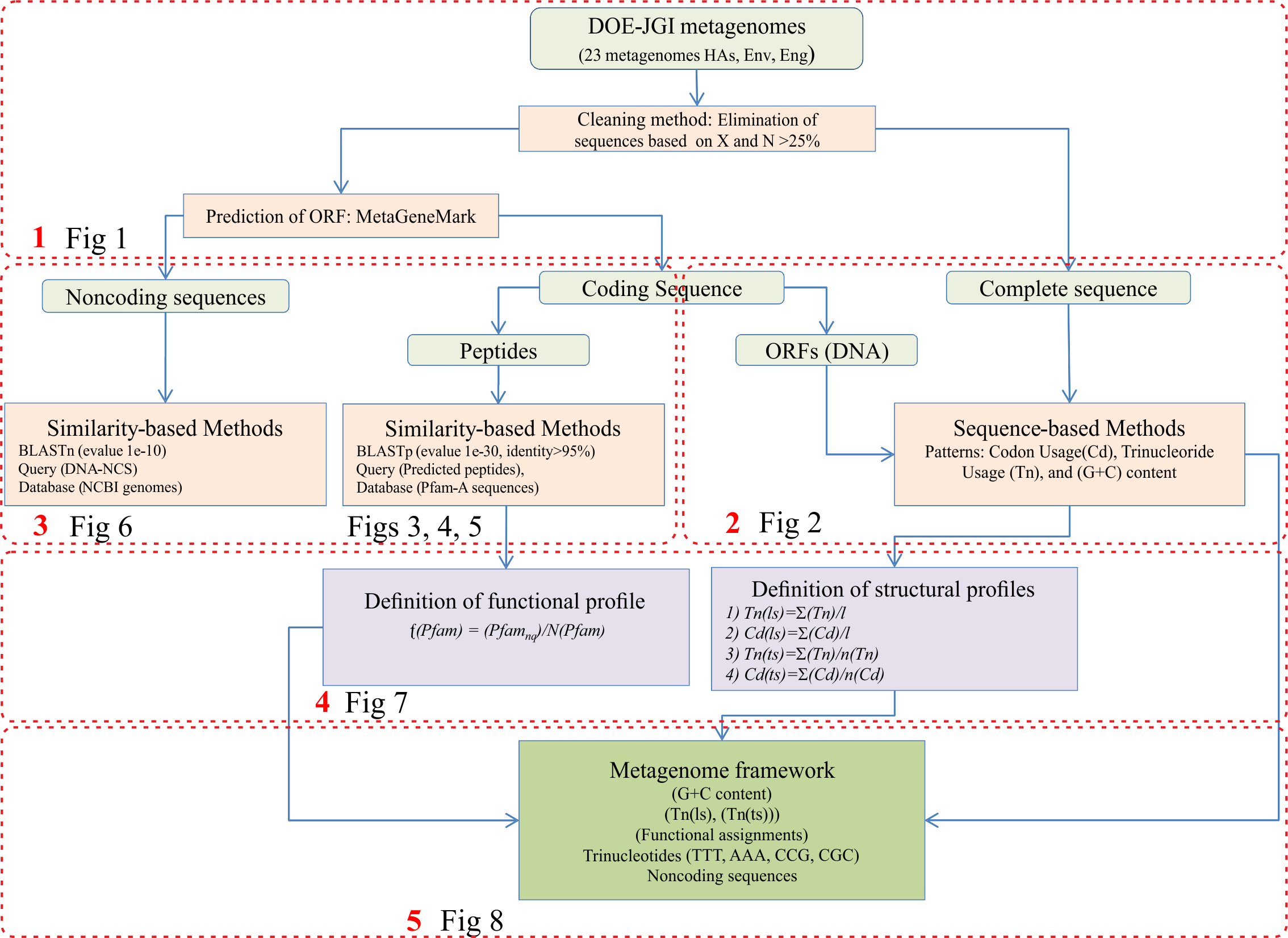

Supplement: Figure S1 — Flowchart of methodological steps. (TIF) [file pone.0059488.s001.tif]
